# Successional and seasonal changes of leaf beetles and their indicator value in a fragmented low thorn forest of northeastern Mexico (Coleoptera, Chrysomelidae)

**DOI:** 10.3897/zookeys.825.30455

**Published:** 2019-02-26

**Authors:** Uriel Jeshua Sánchez-Reyes, Santiago Niño-Maldonado, Shawn M. Clark, Ludivina Barrientos-Lozano, Pedro Almaguer-Sierra

**Affiliations:** 1 Tecnológico Nacional de México-Instituto Tecnológico de Ciudad Victoria. Boulevard Emilio Portes Gil No.1301, C.P. 87010, Ciudad Victoria, Tamaulipas, Mexico Tecnológico Nacional de México Victoria Mexico; 2 Universidad Autónoma de Tamaulipas, Facultad de Ingeniería y Ciencias, Centro Universitario Victoria, C.P. 87149, Ciudad Victoria, Tamaulipas, Mexico Universidad Autónoma de Tamaulipas Victoria Mexico; 3 Brigham Young University, Monte L. Bean Life Science Museum, Provo, Utah 84602, USA Brigham Young University Provo United States of America

**Keywords:** Chronosequence, community patterns, disturbance, seasonality, secondary succession, phytophagous beetles

## Abstract

Leaf beetles (Chrysomelidae: Coleoptera) constitute a highly diverse family of phytophagous insects with high ecological relevance, due to their host plant specificity and their close association to vegetation variables. Therefore, secondary succession and seasonal changes after loss of vegetal cover will have a significant influence on their community patterns. Accordingly, responses of leaf beetles to such environmental heterogeneity make them a suitable taxon for monitoring disturbance, which is more important for endangered habitats such as the low thorn forests (LTF) in northeastern Mexico. We conducted a study in a LTF fragment in order to assess the effects of secondary succession and seasonality on leaf beetle communities, as well as to quantify the importance of Chrysomelidae as an indicator taxon. Landsat scenes were used for delimiting a successional gradient, in which four succession categories were selected: four years, 17 years, and 31 years since loss of vegetal cover, and conserved areas. Eight plots of 100 m^2^ were randomly delimited in each category; plots were sampled monthly, using an entomological sweep net, from May 2016 to April 2017. In total, 384 samples were collected by the end of study, from which 6978 specimens, six subfamilies, 57 genera, and 85 species were obtained. Species richness was higher in early succession areas. Abundance declined significantly from early successional to conserved areas, but the conserved areas had the higher diversity. Furthermore, differences in abundance were significant between rainy and dry seasons in areas of four, 17, and 31 years of succession, but not in conserved areas; also, all categories had a similar abundance during the dry season. Intermediate (17 and 31 years) and conserved areas differed in the season of higher diversity. Regarding inventory completeness, it was close to or above 70 % for all comparisons, although it was very low for the 17-year category during the rainy season. Faunistic similarity was higher between intermediate categories. A total of 24 species had a significant indicator value. Effects of succession time and seasonality on leaf beetle communities are here quantified for first time in LTF forests. Influences of environmental heterogeneity and intermediate disturbance are discussed as main drivers of the results obtained. Several leaf beetle species are proposed that could be useful for monitoring succession time and secondary LTF vegetation in northeastern Mexico. However, studies must be replicated at other regions, in order to obtain a better characterization of disturbance influence on leaf beetles.

## Introduction

Chrysomelidae is the third most diverse family of Coleoptera in the world, with more than 36,000 described species (excluding Bruchinae) (Bouchard et al. 2009). However, recent estimates suggest that this number may be higher, ranging between 55,000 to 60,000 species (Jolivet 2015). This variety resulted from a diversification after the origin of their host plants, as well as from repeated radiations from preexisting diverse plant resources (Gómez-Zurita et al. 2007). Therefore, leaf beetles are almost entirely phytophagous, and their success in ecosystems is determined by their ability to occupy many different feeding niches (Jolivet 1988), and by their host specificity to almost all groups of plants (Riley et al. 2002). Most adults feed on living parts of plants, such as leaves, young stems, flowers, pollen, or fruits (White 1968, Wilcox 1972, Flowers 1996, Riley et al. 2002, Staines 2002, Jolivet and Verma 2008). The larvae are found on the surface of leaves or as leaf miners; others feed on roots, plant litter or submerged parts of plants (White 1968, Riley et al. 2002, Staines 2002, Jolivet and Verma 2008). Some larvae are myrmecophilous and feed on eggs and wastes of ants in their nests (Chamorro-Lacayo 2014). Therefore, leaf beetles are a key group in ecosystems as primary consumers, competing directly with other herbivores (González-Megías and Gómez 2003), and as important components in trophic webs (Basset and Samuelson 1996).

As phytophagous insects, the structure and composition of chrysomelid communities are determined largely by vegetation variables. These include, for example, the type and height of each forest stratum (floristic structure), percentage of vegetation or tree cover, diversity of plants, abundance of young foliage, and specific characteristics of the host plant (Bach 1981, Morrow et al. 1989, Řehounek 2002, Charles and Basset 2005, Baselga and Jiménez-Valverde 2007, Şen and Gök 2009, 2014). In addition, because of their food specificity, leaf beetles are also significantly impacted by modifications in various ecological gradients (Sánchez-Reyes et al. 2015, Sandoval-Becerra et al. 2017), which abiotic factors influence the ability to acquire available resources (Sandoval-Becerra et al. 2018). Therefore, any disturbance as a result of the loss of vegetation cover and land use change will have a direct effect on these insects (Wąsowska 2004).

After disturbance, secondary succession involves subsequent modifications of the vegetation following removal of vegetal cover, and it occurs through different routes, mechanisms, and processes (Pulsford et al. 2016). In turn, these modifications lead to further abiotic and microclimatic changes, which are related to seasonality (Lebrija-Trejos et al. 2011). Despite their importance, the consequences of fragmentation, disturbance, and secondary succession on chrysomelid communities have been quantified only in a few studies (Brown and Hyman 1986, Bach 1990), mainly in temperate wet forests (Linzmeier et al. 2006, Marinoni and Ganho 2006, Linzmeier and Ribeiro-Costa 2009, 2011) and tropical savannas (Pimenta and De-Marco 2015). In addition, in temperate oak forests of northeastern Mexico, the amount of time since last disturbance influences diversity, abundance and spatial distribution of Chrysomelidae (Sandoval-Becerra et al. 2018), as well as their microclimatic niche parameters (Sandoval-Becerra et al. 2017). On the other hand, few works have assessed consistently the effect of seasonality on successional changes of insect communities (Janzen 1976, Linzmeier and Ribeiro-Costa 2009), and some robust studies conducted during several years in areas with different level of conservation in South America, have shown a strong relation between leaf beetles and seasonal changes (Linzmeier and Ribeiro-Costa 2012, 2013). Thus, in order to standardize conservation strategies, it is necessary to evaluate whether the response patterns of leaf beetles to disturbance, secondary succession, and seasonality occur homogeneously in different habitats and ecosystems.

The extent of low thorn forest (LTF) vegetation in northeastern Mexico has been drastically reduced in the last 40 years (Sánchez-Reyes et al. 2017). This ecosystem harbors a high species richness of plants, with a large number of endemics (Rzedowski 2006), and, due to its geographical position, it shares characteristics (presence of deciduous or semi-deciduous species, climate regime) with other important subtropical communities, like the submontane scrub and Tamaulipan thorn scrub (García-Morales et al. 2014). Such floristic complexity presumably leads to a high species richness and diversity of leaf beetles, although this has not been evaluated since the faunistic studies of this family in Mexico are focused in other plant communities (Andrews and Gilbert 2005, Niño-Maldonado et al. 2005, Martínez-Sánchez et al. 2009, Furth 2013, Ordóñez-Reséndiz et al. 2015). In addition, a significant proportion of the current LTF cover in northeastern Mexico is composed of patches of conserved vegetation, together with large areas of secondary vegetation with varying lengths of succession time (Sánchez-Reyes et al. 2017). However, the influences of these disturbances and the time of secondary succession on the faunistic and ecological patterns of Chrysomelidae have never been quantified to date in this ecosystem.

The importance of evaluating changes in leaf beetle communities during secondary succession arises from their potential as indicator taxa. Chrysomelidae is cited as a useful family for monitoring local biodiversity (Farrell and Erwin 1988, Kalaichelvan and Verma 2005, Baselga and Novoa 2007, Aslan and Ayvaz 2009) and quality of the environment (Linzmeier et al. 2006), as well as assessing changes in natural areas (Staines and Staines 2001, Flowers and Hanson 2003). Since leaf beetles are sensitive to environmental modifications in the microhabitat (Sandoval-Becerra et al. 2017), the changes in their community parameters and the presence of certain species could be useful to evaluate ecosystem integrity. However, only a few studies have quantitatively analyzed the indicator value of leaf beetles in the face of habitat changes after disturbance (Pimenta and De-Marco 2015, Sandoval-Becerra 2018). Therefore, the objectives of this study were to: 1) compose a faunistic list of Chrysomelidae in a low thorn forest fragment, 2) compare the species richness, abundance, and diversity in areas with different times of succession, 3) evaluate the seasonal effect in the successional stages, and 4) quantify the indicator value of chrysomelid species to secondary succession.

## Methods

### Study area

**Figure 1.**
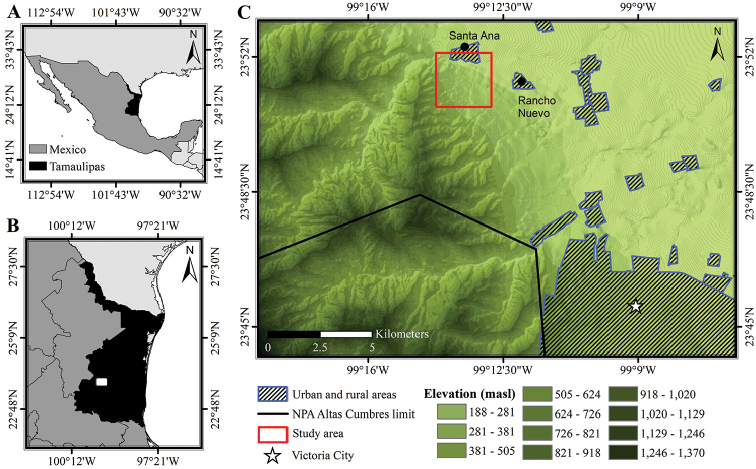
Location of the low thorn forest fragment in northeastern Mexico. **A** Tamaulipas, Mexico **B** location of the fragment within the State **C** detailed location of the LTF fragment in the foothills of the Sierra Madre Oriental, north of the Natural Protected Area Altas Cumbres.

The study was conducted in a LTF fragment, located in the municipality of Victoria in the state of Tamaulipas, northeastern Mexico. In order to rule out the influence of topography on successional community patterns, a specific polygon of approximately 400 hectares was delimited on a plain area with little slope, at a homogeneous elevation between 320 and 350 m a.s.l. (23°51.75'N, 99°14'W and 23°51'N, 99°13.25'W). In addition, the area was located on the eastern foothills of the Sierra Madre Oriental, adjacent to the rural localities of Ejido (Ej.) Rancho Nuevo and Ej. Santa Ana (Figure 1).

Climate is classified as warm subhumid with summer rains, with an average annual temperature between 18 °C and 24.3 °C, and a total annual rainfall of 717.3 to 1058.8 mm (Almaguer-Sierra 2005). The highest volume of precipitation occurs between May and October, although occasional rains of less intensity may occur in other months. Due to this climatic regime, LTF vegetation in northeastern Mexico, particularly in the state of Tamaulipas, can be characterized as deciduous (INEGI 2013) or semideciduous (Treviño-Carreón and Valiente-Banuet 2005). However, there are long drought periods without rain for several months; so, the vegetation could also be classified as a dry forest (Challenger and Soberón 2008), although not with as strongly marked seasonality as that observed in communities of the Mexican Pacific (Ceballos et al. 2010, Trejo 2010). Among the dominant plant species are *Celtispallida* Torr., *Casimiroagreggi* (S. Watson) F. Chiang, *Ebenopsisebano* (Berland.) Barneby & JW Grimes, *Havardiapallens* (Benth.) Britton & Rose, *Randiaobcordata* S Watson, *Cordiaboissieri* A DC, and *Crotoncortesianus* Kunth. In addition, the LTF in the study area is mixed with communities of submontane scrub and Tamaulipan thorn scrub (Treviño-Carreón and Valiente-Banuet 2005, INEGI 2013, García-Morales et al. 2014). In this way, it constitutes a complex plant community of high diversity, with a notable number of endemic plants (García-Morales et al. 2014).

To the southwest, the study area approaches the Natural Protected Area Altas Cumbres (NPAAC, Figure 1), which constitutes a focal point for biodiversity in northeastern Mexico (Morrone and Márquez 2008, Secretaría de Gobierno 2015). However, the distribution of LTF in the areas surrounding the NPAAC, including the studied fragment, has been reduced significantly from 1973 to 2015 due to land use-cover change as a result of frequent fires and conversion to agriculture areas (Sánchez-Reyes et al. 2017), and is currently endangered (Secretaría de Gobierno 2015). Therefore, the study area constitutes a fragment of LTF composed of patches with differing degrees of conservation and times of secondary succession (Sánchez-Reyes et al. 2017).

**Figure 2.**
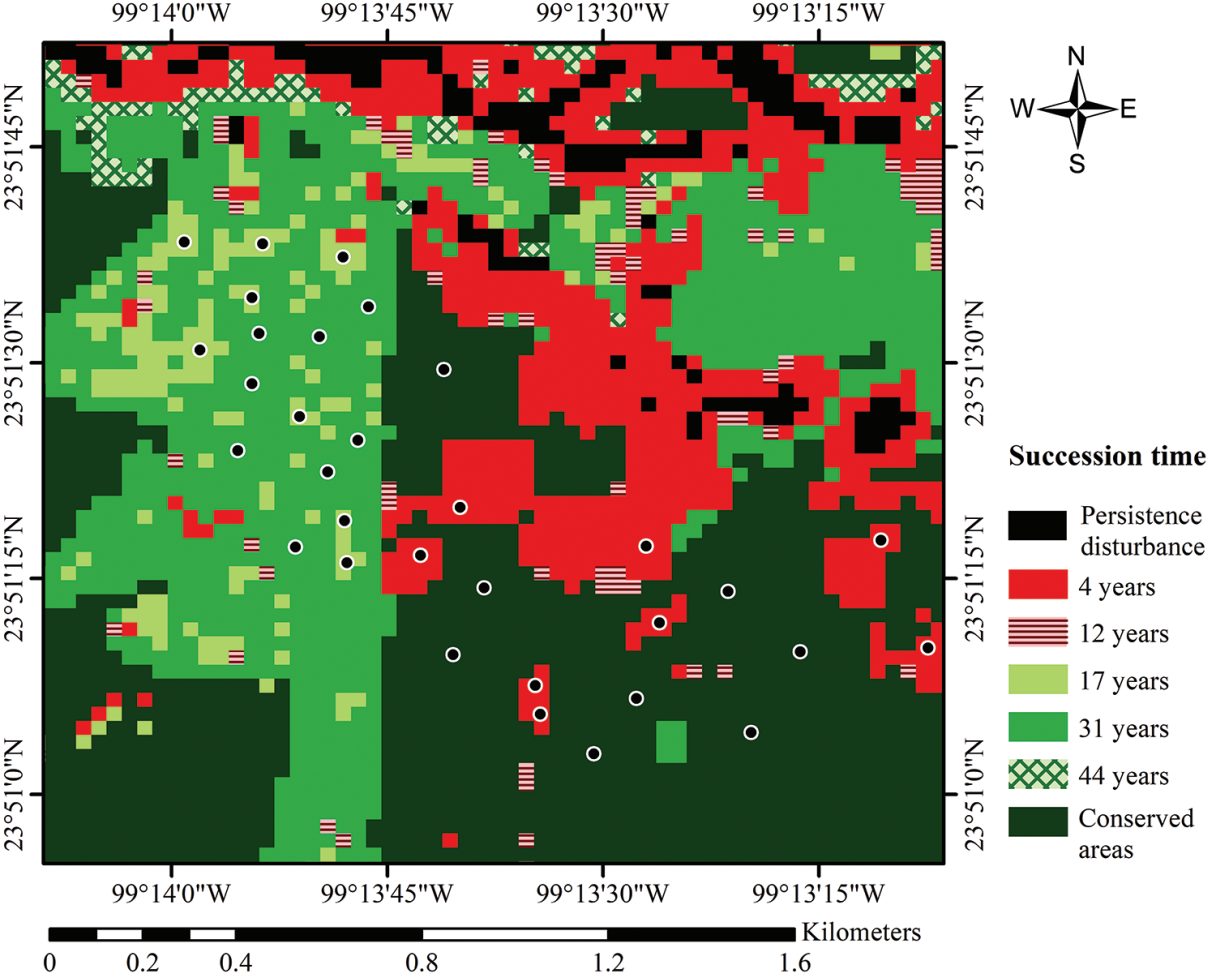
Successional gradient of low thorn forest in northeastern Mexico, and location of the sampling plots.

### Successional gradient delimitation

In the study of secondary succession, it is not always possible to measure the process of modification in a vegetal community over time, usually several years, in the same plot or site (Foster and Tilman 2000, Myster and Malahy 2008). An alternative to evaluate these changes is the use of chronosequences, where the study of succession takes place in space instead of time (Walker et al. 2010). Through this approach, it is assumed, among other factors, that selected sites have the same recovery history and that all succession stages have been developed under similar conditions (Denslow and Guzman 2000, Walker et al. 2010). However, these conditions are rarely considered when succession analysis is performed with chronosequences (Johnson and Miyanishi 2008). The use of geographic information systems and satellite images is an effective strategy to assess these assumptions when quantifying the recovery time on vegetation (Vieira et al. 2003, Arroyo-Mora et al. 2005); so, it can be a useful tool for establishing succession time and delimiting valid chronosequences (Sánchez-Reyes et al. 2017).

We followed the chronosequence approach in this study. Delimitation of succession time of the LTF in the study area was carried out through the analysis of Landsat satellite images of the years 1973, 1986, 2000, 2005 and 2013 (Gutman et al. 2013), employing previously established methodology to calculate the approximate time of succession and define valid chronosequences (Sánchez-Reyes et al. 2017). Briefly, through this method, each of the five images was classified into four types of land use and cover (Table 1), using an unsupervised segmentation, together with the manual selection of training fields and the maximum likelihood algorithm. Afterwards, the four land use and cover categories were unified by means of an image reclassification into only two categories: vegetation and disturbance (Table 1). Finally, the reclassified images were subjected to a cross-tabulation analysis to delimit the time of succession, based on the date since the last disturbance and the transition or persistence of vegetation (LTF) from 1973 to 2013 (Table 2). Complementarily, the current presence of vegetation or disturbance was validated directly in the field during 2016–2017, as well as with recent Google Earth images. In this way, the final succession time in each area was delimited by the changes from 1973 to 2017 (Table 2). Procedures were conducted in the software IDRISI Selva 17.0.

**Table 1.** Land use and cover categories in the study area.

Land cover/land use	Description	Reclassification
Conserved low thorn forest	Primary, conserved vegetation of low deciduous or semi-deciduous thorn forest.	Vegetation
Secondary vegetation of low thorn forest	Secondary arboreal vegetation of low thorn forest; predominance of arboreal species characteristic of submontane scrub and Tamaulipan thorn scrub.	Vegetation
Modified areas	Disturbance areas. Dense or low crop vegetation, active or abandoned agricultural areas, low secondary herbaceous vegetation, grassland cover at ground level.	Disturbance
Bare soil areas	Disturbance areas. Sparse or absent vegetation, dry rivers, rocks, bare soil, rural areas or buildings (human settlements).	Disturbance

**Table 2.** Transitional and persistence processes used to delimit succession categories in the study area. 1 = presence of vegetation (conserved and secondary low thorn forest); 0 = presence of disturbance (modified and bare soil areas).

Category / time of succession	Description	Landsat image					Field validation
		1973	1986	2000	2005	2013	2016, 2017
Conserved areas	Areas with vegetation in 1973 that remained unchanged until 2017	1	1	1	1	1	1
44 years	Areas with disturbance in 1973, but with vegetation in 1986, which persisted until 2017	0	1	1	1	1	1
31 years	Areas with disturbance in 1986, but with vegetation in 2000, which persisted until 2017	–	0	1	1	1	1
17 years	Areas with disturbance in 2000, but with vegetation in 2005, which persisted until 2017	–	–	0	1	1	1
12 years	Areas with disturbance in 2005, but with vegetation in 2017	–	–	–	0	1	1
4 years	Areas with disturbance in 2013, but with vegetation in 2017	–	–	–	–	0	1
Persistence of disturbance	Areas with disturbance in 1973, and remaining unchanged until 2017	0	0	0	0	0	0

### Leaf beetles sampling

Sampling sites were selected according to the extent, location, and accessibility of the successional patches in the study area. Only four categories were selected for this study: 1) areas with four years of succession, 2) areas with 17 years of succession, 3) areas with 31 years of succession, and 4) conserved areas (Figure 2). In each of the four categories, a sample size of eight plots of 10 × 10 m was established; sample size was delimited through the analysis of preliminary data with the Clench model (Jiménez-Valverde and Hortal 2003). Sampling plots were located randomly using a previously established procedure (Sánchez-Reyes et al. 2016) using geographic information systems and specialized software (ArcView GIS, IDRISI Selva 17.0). With this method, it was possible to assess the geographical location of each plot before field sampling, in order to minimize the edge effect, as well as the closeness between plots, which guarantees the feasibility of sampling and the independence of the samples.

Leaf beetles were sampled using an entomological sweep net (60 centimeters long and 40 centimeters in diameter). Each sample consisted of 200 sweeps on all the shrub and herbaceous vegetation in each plot, from soil level up to a maximum height of 2 m, between 10:00 and 14:00 hrs. All contents of the net after 200 sweeps were deposited in a plastic bag, adding 70 % ethyl alcohol, as well as a label with the corresponding data. Each plot of each successional category was sampled once a month, from May 2016 to April 2017, for a total of 384 samples (8 plots x 4 categories x 12 months).

The processing of samples and preparation of specimens were conducted in the laboratory according to previously established methods (Sánchez-Reyes et al. 2014). Taxonomic identification was made using specialized literature (Wilcox 1965, Wilcox 1972, Scherer 1983, White 1993, Flowers 1996, Riley et al. 2002, Staines 2002) and by comparison with specimens in the collection of the Facultad de Ingeniería y Ciencias, Universidad Autónoma de Tamaulipas (FIC-UAT), and identified taxa were organized following the arrangement proposed by Riley et al. (2003) and Bouchard et al. (2011). Specimens that could not be identified to species level were differentiated as morphospecies, based on characteristics of internal genitalia. In this way, the term “species” in this study includes both specimens determined at a specific level and morphospecies. Specimens were deposited in collections of the Instituto Tecnológico de Ciudad Victoria (ITCV) and the FIC-UAT, as well as in the personal collection of the first author (IC-UJSR).

### Data analysis

The observed species richness was measured as the total number of species in the LTF fragment, as well as at each successional category. Significant differences in the number of species among categories were determined by the diversity permutation test implemented in PAST 3.07 (Hammer et al. 2001). Estimated species richness was calculated for the entire study area and for each category, using the Chao 1, Chao 2, Jackknife 1, Jackknife 2, ACE and ICE nonparametric indices. Inclusion of these indices is recommended in biodiversity studies to evaluate the estimated range of species in the faunistic inventory (Hortal et al. 2006); calculations were made in EstimateS 8.2 (Colwell 2013), using 100 randomizations without replacement. We also calculated the estimated species richness through the Clench model, as well as the slope value, in order to determine the quality of inventories, where values close to or less than 0.1 are considered characteristic of reliable inventories (Jiménez-Valverde and Hortal 2003). The Clench model was performed in STATISTICA 8.0 (StatSoft Inc. 2007), following the parameters indicated by Jiménez-Valverde and Hortal (2003). Completeness of the total inventory and of each category was obtained as the proportion of observed richness with respect only to the estimated value of the Chao 1 index, since it takes into account the abundance of species and acts as a lower bound for species richness (Chao and Chiu 2016), and was expressed as percentage.

Overall differences in abundance of leaf beetle communities in the successional gradient were calculated with the Kruskal-Wallis test, after discarding a normal distribution. In addition, significant differences in abundance between categories were obtained through pairwise comparisons, using the Mann-Whitney test. Diversity was considered as a proportional value between species richness and abundance values, and was quantified by Simpson’s dominance index and the Shannon entropy index (Magurran 2004); these were calculated for the entire study area and for each successional category. Pairwise comparisons of diversity values ​​between categories were carried out using the diversity permutation test. Changes in species composition between successional categories were evaluated by comparing faunistic similarity, using the Bray-Curtis index (Magurran 2004). All analyzes were performed in PAST 3.07.

Seasonal effect was measured separately, by comparing observed and estimated species richness, abundance, and diversity in each category during the rainy (May to October 2016) and dry seasons (November 2016 to April 2017); differences in abiotic conditions between both seasons were confirmed in a previous study (Sánchez-Reyes et al. in press). The aforementioned indices and statistical tests were used for such comparisons: nonparametric estimation of species richness, Kruskal-Wallis and Mann-Whitney tests for differences in abundance, diversity permutation tests for observed species richness, and Simpson and Shannon indices, which were conducted in PAST 3.07. An agglomerative Cluster analysis was performed in order to include the seasonal effect in faunistic composition, with the objective of grouping categories and seasons according to their similarity in species composition. For this, a dissimilarity matrix based on Bray-Curtis distance and Ward’s amalgamation algorithm was used. Cluster analysis was performed in STATISTICA 8.0.

The indicator value of chrysomelid species was quantified by the Indicator Value Index, or IndVal (Dufrêne and Legendre 1997). The index is based on the degree of specificity (exclusivity of the species to a particular site based on its abundance) and the degree of fidelity (frequency of occurrence within the same habitat) (Tejeda-Cruz et al. 2008), expressed in a percentage value. The analysis was carried out using the *labsdv* package in platform R version 3.2.2, using 1000 random permutations to define the significance level. Indicator species with an index equal to or greater than 70 % were categorized as “characteristic” species, while species with a value less than 70 % but equal to or greater than 30 % were considered “detector” species.

## Results

### Successional variation of leaf beetle communities

In total, 6978 specimens of leaf beetles were collected distributed in six subfamilies, 57 genera, and 85 species (Appendix 1). The greatest abundance and species richness were observed in the subfamily Galerucinae (6416 specimens and 40 species respectively). Total values of estimated richness in the low thorn forest fragment suggested a reliable inventory (slope = 0.029), with a total completeness of 82.52 %. The dominance was 0.284, while the Shannon index was 2.009 (Table 3).

**Table 3.** Succession parameters of leaf beetle communities in a low thorn forest in northeastern Mexico.

Ecological parameter	Low thorn forest total	Succession time			
		4 years	17 years	31 years	Conserved areas
Observed richness*	85	58 a	36 b	31 b	45 ab
Chao 1	103	70.07	66.23	33.57	52.55
Chao 2	97.86	72.07	53.81	34.96	56.95
Jackknife 1	103.95	73.83	47.88	38.92	57.86
Jackknife 2	108.96	80.78	55.75	39	63.81
ICE	101.34	71.4	44.43	37.63	58.64
ACE	99.91	68.05	44.52	34.9	52.43
Clench model	95.53	68.40	46.32	40.27	55.16
Slope	0.029	0.103	0.087	0.075	0.093
Completeness (%)	82.52	82.77	54.35	92.34	85.63
Abundance*	6978	2725 a	1753 a	1674 ab	826 b
Dominance (Simpson index)*	0.2841	0.2174 a	0.6543 b	0.781 c	0.1469 d
Diversity (Shannon index)*	2.009	2.084 a	0.9797 b	0.6796 c	2.521 d

*Different letters between columns are significantly different from each other.

The analysis of successional categories revealed that the species richness was significantly higher in the areas of four years of succession. The conserved areas also demonstrated a high number of species, although the value was similar to that observed in the intermediate categories (17 and 31 years of succession). In all categories, the inventories were reliable according to the Clench slope values, and the observed species richness values were close to the estimated values; areas of 4 and 31 years of succession, as well as conserved areas, had a completeness value above 70 %. However, a low value of completeness (54.35 %) was obtained only in the areas of 17 years of succession (Table 3). The abundance decreased significantly with the increase in time of succession (H = 12.56, *p* = 0.005). Thus, early succession sites had the highest number of specimens when compared to the conserved areas. The values ​​of diversity were significantly different among all the categories (*p* < 0.05). Highest dominance and lowest diversity were obtained in the intermediate succession areas (17 and 31 years); in contrast, the conserved areas recorded the highest diversity value (Table 3).

With respect to the Bray-Curtis index, a very high similarity was observed in the faunistic composition between the intermediate (17 and 31 years) successional areas (94.56 %). Remaining comparisons were below 50 % similarity (Table 4).

**Table 4.** Faunistic similarity of Chrysomelidae between successional categories of a low thorn forest in northeastern Mexico. Upper diagonal, values of the Bray-Curtis index. Lower diagonal, values expressed in percentage.

	4 years	17 years	31 years	Conserved areas
**4 years**	1	0.3135	0.2629	0.2225
**17 years**	31.35 %	1	0.9456	0.2300
**31 years**	26.29 %	94.56 %	1	0.2265
**Conserved areas**	22.25 %	23 %	22.65 %	1

### Effect of seasonality on the successional changes of Chrysomelidae

**Figure 3.**
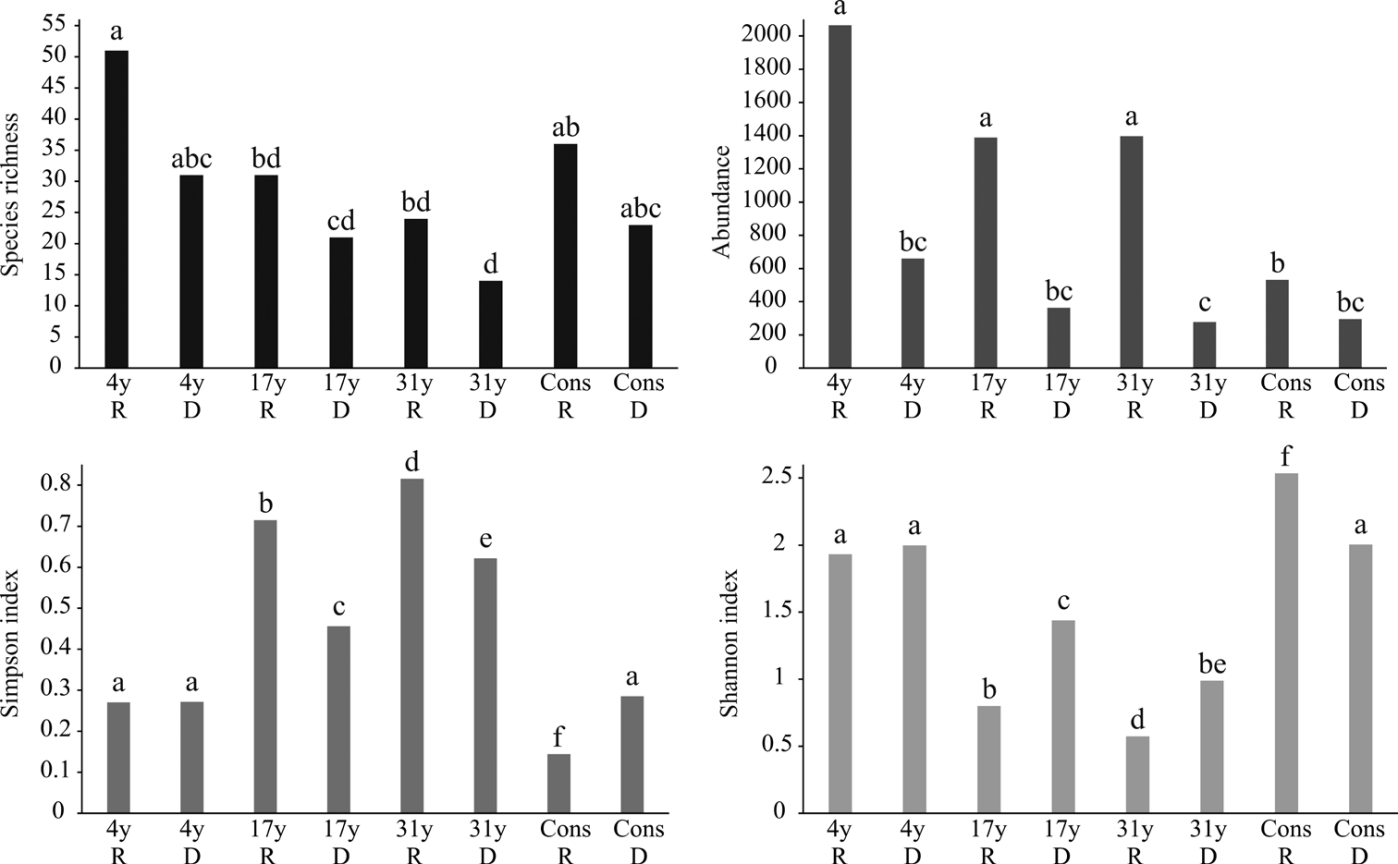
Seasonal variation of community parameters of Chrysomelidae in a successional gradient of low thorn forest in northeastern Mexico. Different letters between bars indicate significant differences.

Seasonal effect was absent in the observed species richness, since there were no significant differences (*p* > 0.05) between the rainy and dry seasons in any of the four categories (Figure 3). The estimated richness analysis revealed that both inventory reliability and completeness were higher in the dry season than in the rainy season, in all categories (Table 5). On the other hand, in all cases the completeness was higher than 70 %; however, in the 17-year category, the estimated richness reached very high proportions during the rainy season, with a total completeness of 35.54 % (Table 5).

**Table 5.** Influence of seasonality on the estimated species richness and inventory completeness of successional categories in a low thorn forest in northeastern Mexico.

	4 years		17 years		31 years		Conserved	
Estimator index	Rainy	Dry	Rainy	Dry	Rainy	Dry	Rainy	Dry
Chao 1	70.59	31.64	87.21	21.07	27.12	14.1	42.39	23.9
Chao 2	71.21	31.37	66.37	21.05	32	14.06	52.32	23.63
Jack 1	67.65	33.94	47.65	21.98	30.85	14.98	45.79	25.94
Jack 2	77.37	25.56	60.19	13.56	34.75	8.43	52.56	22.25
ICE	68.37	32.09	66.59	21.36	29.21	14.4	44.56	24.6
ACE	63.47	32.35	57.91	21.36	27.45	14.47	42.27	24.82
Clench model	63.12	38.40	47.94	27.51	30.67	18.80	43.22	28.69
Slope	0.222	0.121	0.233	0.096	0.111	0.071	0.136	0.091
Completeness (%)	72.24	97.97	35.54	99.66	88.49	99.29	84.92	96.23

Differences in abundance between the rainy and dry season were significant in each category (*p* < 0.05), except for the conserved areas where the number of specimens was similar in both seasons (*p* = 0.0904). In addition, during the rainy season, the abundances observed in the areas of 4, 17, and 31 years were similar, but significantly different from the conserved areas; contrarily, during the dry season there were no differences in abundance between categories (Figure 3). Regarding diversity, the seasonal effect was not observed in the areas of 4 years of succession, since there were no differences in either the Simpson or Shannon indices between seasons. In the case of the intermediate areas (17 and 31 years of succession), lower dominance and higher diversity occurred during the dry season. Conversely, the minimum dominance and maximum diversity values in the study area were obtained in the conserved areas during the rainy season (Figure 3).

**Figure 4.**
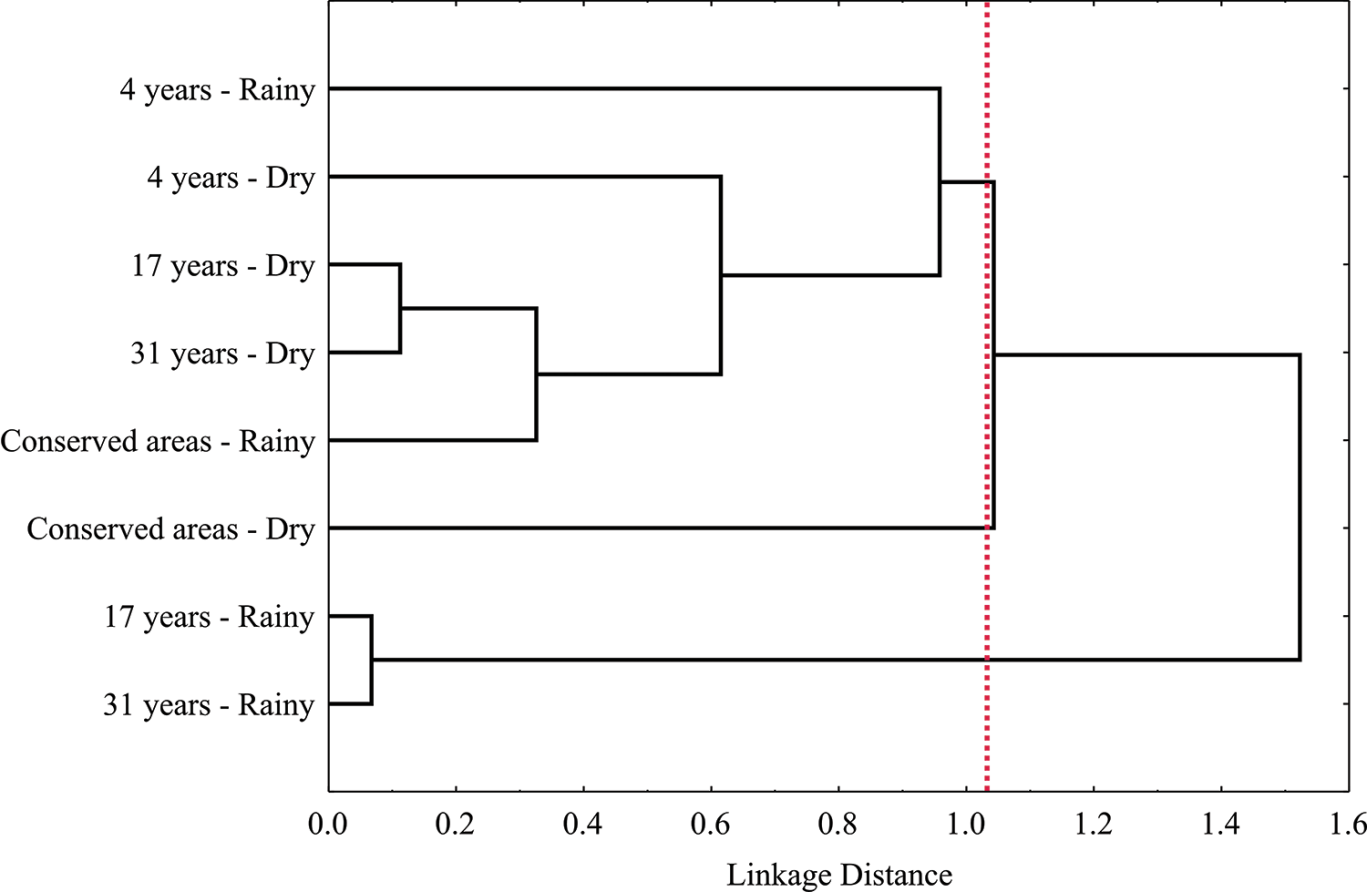
Cluster analysis of leaf beetle composition between succession and seasonal categories (rainy and dry) in a low thorn forest in northeastern Mexico. The dotted line indicates the delimitation of the groups.

The analysis of similarity in species composition between successional categories, considering the seasonal effect, suggested the presence of three faunistic groups. The first group consisted of the areas of 17 and 31 years of succession during the rainy season. Conserved areas during the dry season represented another group. The category of four years of succession, intermediate areas during the dry season, and conserved areas in the rainy season formed a third group with higher heterogeneity (Figure 4).

### Indicator value of leaf beetles in the successional gradient

**Figure 5.**
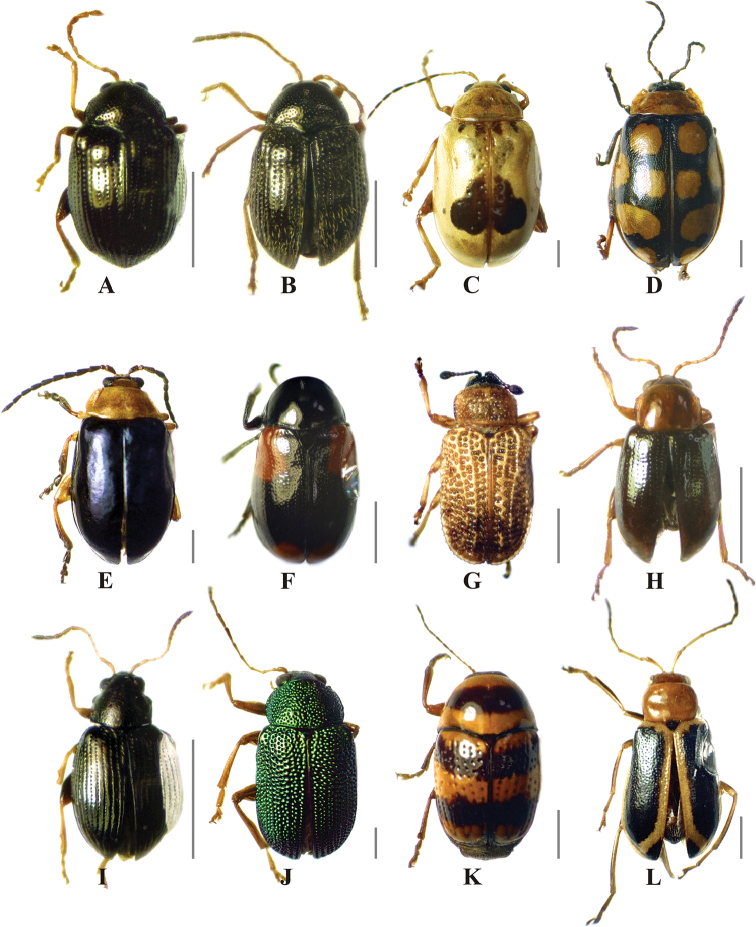
Chrysomelidae species with significant indicator value of successional time in a low thorn forest fragment in northeastern Mexico. **A***Acallepitrix* sp. 1 **B***Epitrix* sp. 5 **C***Acrocyumdorsalis* Jacoby, 1885 **D***Alagoasajacobiana* (Horn, 1889) **E***Asphaera* sp. 1 **F***Babiatetraspilotatexana* Schaeffer, 1933 **G***Brachycorynapumila* Guérin-Méneville, 1844 **H***Centralaphthonadiversa* (Baly, 1877) **I***Chaetocnema* sp. 1 **J***Colaspistownsendi* Bowditch, 1921 **K***Cryptocephalustrizonatus* Suffrian, 1858 **L***Cyclotrypemafurcata* (Olivier, 1808). Scale bar: 1 mm.

**Figure 6.**
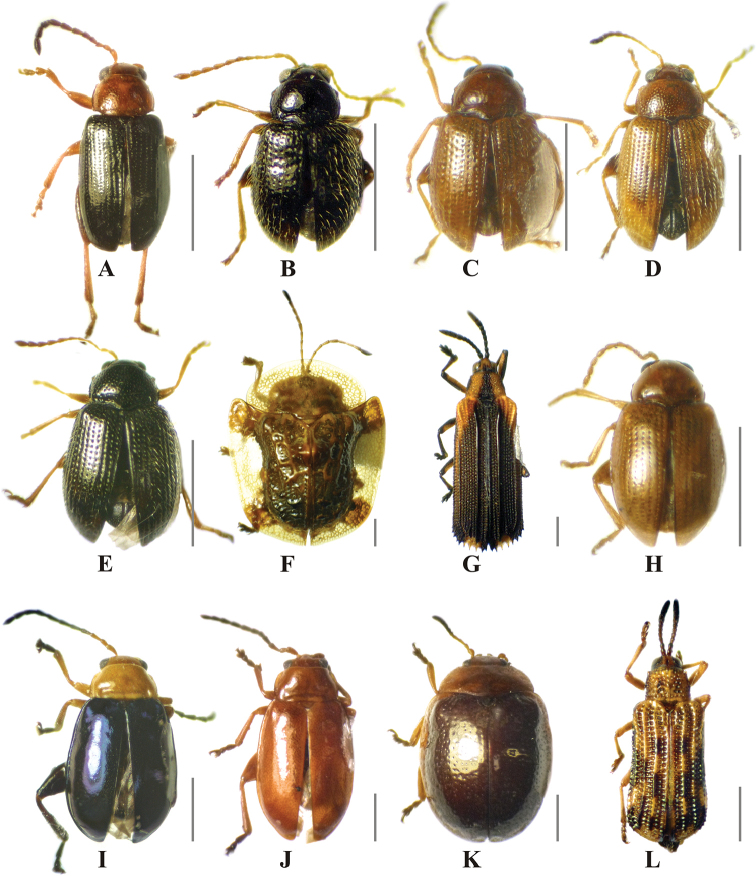
Chrysomelidae species with significant indicator value of successional time in a low thorn forest fragment in northeastern Mexico. **A***Dysphenges* sp. 1 **B***Epitrix* sp. 1 **C***Epitrix* sp. 2 **D***Epitrix* sp. 3 **E***Epitrix* sp. 4 **F***Helocassisclavata* (Fabricius, 1798) **G***Heterispavinula* (Erichson, 1847) **H***Margaridisa* sp. 1 **I***Parchicola* sp. 1 **J***Parchicola* sp. 2 **K***Plagioderathymaloides* Stål, 1860 **L***Sumitrosisinaequalis* (Weber, 1801). Scale bar: 1 mm.

Of the 85 total species found in the LTF, only 24 had a significant indicator value (*p* < 0.05, Table 6, Figures 5, 6). The highest proportion involved detector species, with an IndVal between 30 and 70 % (17 species). The remaining seven were characteristic species, with values higher than 70 % (Table 6). Four species were considered as characteristic of areas with four years of succession, of which *Brachycorynapumila* Guérin-Méneville, 1844 and *Chaetocnema* sp. 1 had the highest indicator values; the other six species were detectors of this category. *Cyclotrypemafurcata* (Olivier, 1808) and *Heterispavinula* (Erichson, 1847) were detector species of areas with 17 years of succession, while *Centralaphthonadiversa* (Baly, 1877), *Dysphenges* sp. 1 and *Sumitrosisinaequalis* (Weber, 1801) were detectors of 31 years of succession. In the conserved areas, six species were detectors; only *Acrocyumdorsalis* Jacoby, 1885, *Margaridisa* sp. 1 and *Parchicola* sp. 1 were characteristic indicators, the latter being the one with the highest specificity.

**Table 6.** Leaf beetle species with a significant indicator value in a successional gradient of low thorn forest in northeastern Mexico. Indicator values in succession categories are expressed in percentage. Key: C = characteristic; D = Detector, *p* = probability.

Species	Succession time				*p*	Indicator category
	4 years	17 years	31 years	Conserved areas		
*Acallepitrix* sp. 1	0.00	7.89	0.66	47.37	0.0048	D
* Acrocyum dorsalis *	0.00	0.00	0.00	75.00	0.0003	C
* Alagoasa jacobiana *	4.46	2.38	5.36	56.25	0.0017	D
*Asphaera* sp. 1	34.09	1.14	0.00	0.00	0.0433	D
* Babia tetraspilota texana *	53.85	7.21	11.54	0.48	0.0021	D
* Brachycoryna pumila *	88.73	2.82	0.00	0.00	0.0001	C
* Centralaphthona diversa *	11.08	40.83	42.74	5.35	0.0241	D
*Chaetocnema* sp. 1	93.47	5.55	0.00	0.02	0.0001	C
* Colaspis townsendi *	53.35	0.30	0.00	3.05	0.0072	D
* Cryptocephalus trizonatus *	37.50	0.00	0.00	0.00	0.0492	D
* Cyclotrypema furcata *	30.65	55.91	0.81	0.00	0.0047	D
*Dysphenges* sp. 1	21.88	14.51	42.19	0.45	0.0282	D
*Epitrix* sp. 1	90.87	0.15	0.02	5.24	0.0001	C
*Epitrix* sp. 2	19.44	4.86	0.52	63.89	0.0001	D
*Epitrix* sp. 3	75.38	0.34	0.10	3.89	0.0003	C
*Epitrix* sp. 4	7.34	0.27	0.00	58.70	0.0036	D
*Epitrix* sp. 5	1.97	7.89	0.00	39.47	0.0327	D
* Helocassis clavata *	5.07	2.70	0.68	61.49	0.0014	D
* Heterispa vinula *	7.50	45.21	18.33	0.97	0.0351	D
*Margaridisa* sp. 1	12.74	0.12	1.14	72.96	0.0002	C
*Parchicola* sp. 1	1.32	0.00	0.00	78.29	0.0001	C
*Parchicola* sp. 2	37.50	0.00	0.00	0.00	0.0483	D
* Plagiodera thymaloides *	42.86	0.45	19.64	0.00	0.0257	D
* Sumitrosis inaequalis *	0.83	5.00	36.67	0.00	0.0353	D

## Discussion

The present study constitutes the first faunistic contribution of Chrysomelidae in the low thorn forest vegetation. The observed species richness and completeness values​​ suggest that the fauna of leaf beetles in the LTF is close or superior to other types of low tropical forest (Sánchez-Reyes et al. 2014, Lucio-García 2018). In general terms, the values represent 34 % of the observed chrysomelid richness in Tamaulipas (Niño-Maldonado et al. 2014), as well as 3.19 % of the country-wide values (Niño-Maldonado and Sánchez-Reyes 2017). In addition, the abundance values were higher than for other studies carried out in the region, including those conducted in larger geographical areas also during an annual period (Niño-Maldonado et al. 2005, Sánchez-Reyes et al. 2014, 2016). One of the factors that can give rise to such results is the complexity of the plant structure in the study area, which consists of tropical vegetation elements in conjunction with species from semi-arid or subtropical areas, such as Tamaulipan thorn scrub or submontane scrub (Treviño-Carreón and Valiente-Banuet 2005, García-Morales et al. 2014). This floristic complexity was mirrored in the presence of an equally complex chrysomelid fauna. In addition, the proximity to the NPA Altas Cumbres (Secretaría de Gobierno 2015) and the adjacent location of one of the panbiogeographic nodes of Mexico (Morrone and Márquez 2008) should undoubtedly be related to the species composition observed.

However, the environmental heterogeneity in the LTF, resulting from disturbance and secondary succession, is perhaps the most important factor leading to the observed patterns. Previous studies had been carried out mainly in natural protected areas, where ecosystems have a high degree of conservation and low occurrence of fragmentation (Niño-Maldonado et al. 2005, Sánchez-Reyes et al. 2014, 2016). Contrastingly, LTF in northeastern Mexico, particularly in the eastern boundaries of the NPA Altas Cumbres, constitutes a highly heterogeneous habitat, which has been subject to strong fragmentation and presently is composed of patches with different succession times (Sánchez-Reyes et al. 2017). Environmental heterogeneity is one determining factor in the structure of communities, and, although it can be variable, its increase is positively related to the species richness (Tamme et al. 2010, Stein et al. 2014, Yang et al. 2015). Such an effect has been observed in other studies of Chrysomelidae; for example, in temperate forests of northeastern Mexico, a high diversity is associated with a heterogeneous mosaic of vegetation with different times since last disturbance (Sandoval-Becerra et al. 2018). In that sense, the influence of heterogeneity was made evident in this study through the detailed comparisons of species richness, abundance, and diversity between areas with different times of succession.

Responses of communities to disturbance are diverse (Winter et al. 2015). Overall, in the LTF the greatest number of species occurred in the category of 4 years of succession. Early successional areas (those without dense tree cover or with open canopy) can promote diversity due to their high structural and spatial complexity (Swanson et al. 2011). On the other hand, the estimators were very close to the observed richness for each successional category, indicating that faunistic inventory was nearly complete, and thus that the calculated parameters are reliable. Several studies in disturbance-succession gradients found that the greatest number of insects occurred in advanced stages of recovery, or in mature, conserved areas (Barberena-Arias and Aide 2003, Jeffries et al. 2006, Villa-Galaviz et al. 2012, Perry et al. 2016). However, this depends on the ecological characteristics of the taxa analyzed, since disturbance can contribute to the increase in diversity (Steffan-Dewenter and Tscharntke 2001, Stephen and Sánchez 2014, Winter et al. 2015, Yuan et al. 2016, O’Brien et al. 2017). In the case of leaf beetles, it has been observed that species richness augments in modified areas (Heusi-Silveira et al. 2012), as was observed in this study. This is because, in recently disturbed or early succession areas, the density of herbaceous and shrub plants is usually higher. This favors the presence of a greater number of phytophagous insects in these areas, given their close association with successional changes of vegetation (Fernandes et al. 2010, O’Brien et al. 2017). In addition, in the area of this study, the early succession stages of LTF are also dominated by species from other plant communities, such as submontane scrub or Tamaulipan thorn scrub (Canizales-Velázquez et al. 2009). Therefore, early succession areas constitute habitats of a complex floristic structure, which triggers a higher environmental heterogeneity and thus explains the high number of chrysomelid species. With respect to abundance, the obtained results agree with trends observed in other studies, since the number of specimens decreased linearly from early succession to the most conserved areas (Heusi-Silveira et al. 2012, O’Brien et al. 2017). Similarly, this is attributed to a higher density of herbaceous and shrub plants in recently disturbed areas (Guariguata and Ostertag 2001, Pickett et al. 2008, Swanson et al. 2011). Besides, this decline in abundance was related to a drop in dominance values in conserved areas, in a way that this category registered the highest diversity, that is, the highest effective number of species considering an equitable community (Jost 2006). Over the course of succession, increase in stability of communities occurs, while fluctuations in availability of resources for species decrease (Anderson 2007). This homogeneity in environmental conditions allows for an even distribution in the relationship between species richness and abundance, thus increasing diversity (Magurran 2004).

Seasonality had a very important influence on the successional patterns. However, differences in species richness were non-existent between seasons. Thus, the number of leaf beetle species was similar throughout the year in each successional category. On the other hand, the number of specimens was significantly higher during the rainy season in all successional categories (4, 17, and 31 years); contrarily, no differences were observed in conserved areas. This is attributed to the vegetation characteristics in successional areas. An open structure of the canopy allows for a greater light input, which during the rainy season favors a high density of annual herbaceous and shrub species (Guariguata and Ostertag 2001, Pickett et al. 2008, Swanson et al. 2011), which constitute a very abundant food resource, but only during one season. Over the course of succession, dominance of shrubs and other perennial herbaceous plants increases (Swanson et al. 2011); indeed, the understory in conserved areas of LTF is dominated mostly by perennial or semiperennial species (Treviño-Carreón and Valiente-Banuet 2005, García-Morales et al. 2014), and these satisfy nourishing requirements of leaf beetles even during the dry season. Such an effect of transition from annual to perennials plants during succession has also been observed in other groups of insects (Steffan-Dewenter and Tscharntke 2001).

With respect to diversity, the intermediate categories (17 and 31 years of succession) showed significantly higher values in the dry season, while in the conserved areas they were higher during the rainy season. Intermediate areas of LTF are spatially and floristically heterogeneous; thus, the existence of annual species from other scrub communities must be responsible for the drastic reduction of understory vegetation in the dry season; consequently, this causes the decrease in abundance, reducing dominance and increasing diversity. Oppositely, the environmental conditions during the rainy season in conserved areas seem to be supporting a greater availability of resources, in such a way that leaf beetle species are uniformly dispersed; this can be attributed to the higher specialization of species in mature or conserved areas (Pellissier 2015). In contrast, the absence of differences in seasonal diversity at early succession areas can be attributed to generalist species, which are dominant in highly heterogeneous areas (Büchi and Vuilleumier 2014). Consequently, dominance and diversity remain constant throughout the year, regardless of available resources in each season, since generalists feed on multiple plant species and tolerate a wide range of microclimatic conditions (Sandoval-Becerra et al. 2018). An example of such species is *Brachycorynapumila*, because its abundance was higher in areas of four years of succession in both seasons, thus suggesting a wide abiotic tolerance; also, *B.pumila* is associated with several species of plants in different genera, such as *Abutilon*, *Malvastrum*, or *Sida*, among others (Staines 1986). However, biological and ecological information is lacking for most of the leaf beetle species in the region; therefore, future assessments on niche requirements of leaf beetles need to be conducted.

In addition to the consequences to abundance and diversity, disturbance and successional changes also influence species composition (Perry et al. 2016). For example, it has been observed that dry season results in greater faunistic similarity between different sites, despite having different degrees of conservation (Janzen 1976). Such an influence was evident in this work, since, according to the Cluster analysis, the areas of 4, 17, and 31 years had the same species composition during the dry season. Besides, the conserved areas had a totally different composition with respect to the other successional stages, which agrees with patterns observed in several other insect taxa (Barberena-Arias and Aide 2003, Perry et al. 2016). In this regard, early succession areas and high heterogeneity due to disturbance may indeed increase the number of species, as was discussed above; however, the similarity of faunistic composition between such areas and conserved sites is very low. Thus, it is necessary to point out the importance of the conserved areas for Chrysomelidae distribution in the low thorn forest vegetation, as their communities are unique to these types of habitats when compared with disturbed areas.

An important theory that aims to explain the relationship between disturbance and diversity is the intermediate disturbance hypothesis (IDH, Connell 1978, Willig and Presley 2018). It postulates that with the increase of the disturbance an imbalance is created in the environmental conditions, and that this reduces the probability of exclusion among coexisting species (Connell 1978, Huston 2014). Thus, intermediate areas represent a convergence of both ends of the gradient, promoting greater heterogeneity in conditions that allows an increase in the resource availability, as well as in the richness and diversity of species (Shea et al. 2004, Bongers et al. 2009, Roxburgh et al. 2004, Winter et al. 2015). Also, intermediate areas may increase herbivory patterns (Chapin et al. 2011). The influence of IDH has been proven previously with Chrysomelidae in other types of vegetation (Linzmeier et al. 2006, Linzmeier and Ribeiro-Costa 2009, Sandoval-Becerra et al. 2018). Observed results in the LTF were contrary, since as explained above, the intermediate areas of 17 and 31 years of succession had very low values ​​of richness and diversity. However, richness estimators can determine the validity of the IDH in the study area, because the category of 17 years of succession could reach above 87 species during the rainy season, while the completeness was higher in the other categories and seasons. Such an estimated value was close to the total observed richness for the entire LTF fragment, confirming that areas of 17 years are a heterogeneous environment with characteristics of both early and late successional stages, creating niches for numerous species (Sandoval-Becerra et al. 2018). It is possible that the randomized design of the study caused that location of most of the intermediate sampling plots fell in areas with low density of herbaceous and shrub vegetation, while only a few plots had contrasting conditions. Such differences would be enhanced due to a higher environmental heterogeneity in the rainy season. In addition, the convergence of species from early and late succession areas was supported by the higher faunistic similarity observed between the categories of 17 and 31 years of succession, even when including a seasonal effect. Therefore, it is fairly possible that community patterns of Chrysomelidae in the LTF are consistent with IDH, although future studies with a higher number of samples are necessary to corroborate this presumption.

Overall, the seasonal effect on leaf beetle communities has been previously assessed in natural gradients (Bouzan et al. 2015, Sánchez-Reyes et al. 2014, 2016). However, the interaction between seasonality and secondary succession, as investigated here, has been poorly quantified (Janzen 1976, Linzmeier and Ribeiro-Costa 2009). Analysis of these patterns is important to determine the magnitude of changes in insect communities after disturbance, so that conservation strategies can be applied successfully. Besides, the understanding of seasonal influence on successional trajectories is critical not only for leaf beetles, but also for other biological groups, since incoming environmental modifications due to climate change surely will affect the mechanisms in which communities response to disturbance, and will therefore affect their resilience aptitude. Hence, more studies are sorely needed in order to clarify these associations, since the responses to succession and disturbance are influenced by microenvironmental changes, and these responses differ depending on the species (Sandoval-Becerra et al. 2017).

Regarding the indicator potential of Chrysomelidae, other studies have suggested that the higher proportion and abundance of leaf beetles are characteristics of early succession or recently disturbed areas (Pimenta and De-Marco 2015), which was corroborated in this study. However, it is evident that the observed community patterns (such as abundance) are driven by the specific response of each species, since leaf beetles can tolerate different microclimatic and microhabitat conditions after disturbance (Sandoval-Becerra et al. 2017). Therefore, these characteristics may allow species within this family to be used as indicators. The potential of leaf beetles for environmental monitoring has been suggested by other authors (Nummelin and Borowiec 1992, Flowers and Hanson 2003, Linzmeier et al. 2006). Similarly, the usefulness of chrysomelid diversity as an indicator of plant species richness has been recognized (Kalaichelvan and Verma 2005, Baselga and Novoa 2007). However, only a few exceptional studies have presented quantifiable indicator values (Pimenta and De-Marco 2015).

**Figure 7.**
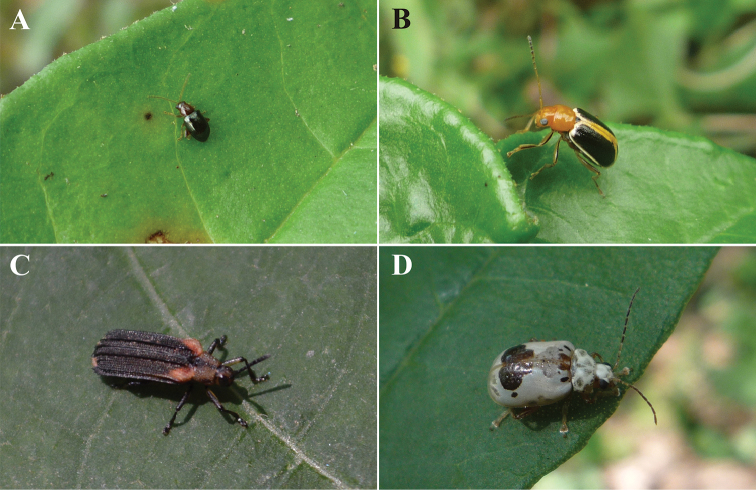
Suggested species of Chrysomelidae for evaluating successional time and environmental monitoring of low thorn forest in northeastern Mexico. **A***Centralaphthonadiversa* (Baly, 1877) **B***Cyclotrypemafurcata* (Olivier, 1808) **C***Heterispavinula* (Erichson, 1847) **D***Acrocyumdorsalis* Jacoby, 1885.

The indicator value index (IndVal) allows for statistical evaluation of the degree of association between species and their environment (Dufrêne and Legendre 1997, De-Cáceres et al. 2012). Species with a significant IndVal can be separated into characteristic and detector species, according to their specificity towards a particular habitat condition. The characteristic indicator species are those with high specificity and fidelity to a given habitat, and therefore a high percentage of indicator value (Dufrêne and Legendre 1997, McGeoch et al. 2002, Tejeda-Cruz et al. 2008). These may be important from an ecological perspective, but not very useful for disturbance quantification, since their presence is exclusive of certain habitats and thus the destruction or modification of such areas would drive local extinction (Hodkinson and Jackson 2005). In contrast, indicator species with moderate levels of specificity (i.e., detector species) have differing degrees of preference for the various ecological states (Dufrêne and Legendre 1997, McGeoch et al. 2002, Tejeda-Cruz et al. 2008). Such detector species are ideal for monitoring, because their wide niche breadth and their changes in abundance during disturbance ensure that they can be dispersed along a disturbance gradient (McGeoch et al. 2002, Hodkinson and Jackson 2005). Accordingly, we consider that indicator species in this study, such as *Centralaphthonadiversa*, *Cyclotrypemafurcata* and *Heterispavinula*, are potentially suitable as indicators of succession time and for environmental monitoring in other areas with similar vegetation; in addition, species like *Acrocyumdorsalis* could be useful to detect conserved areas of LTF (Figure 7). These species are easily identifiable (even in the field), are abundant (so their proportion in different degrees of disturbance can be statistically quantified), and are widely distributed in the region (Niño-Maldonado et al. 2014, Sánchez-Reyes et al. 2016). Therefore, they fulfill the expected characteristics for a good indicator taxon (Carignan and Villard 2002, Ribera and Foster 1997). Besides, their response as significant indicator species is consistent with other ecological gradients, such as microclimate or elevation (Sánchez-Reyes et al. 2016, 2017). However, their presence as an indicator in this study does not guarantee that their response to succession time will be homogeneous in other areas; such responses must be consistent and repeatable in different sites and time scales in order to consider a species as a reliable indicator (McGeoch et al. 2002, Hodkinson and Jackson 2005). Currently, we suggest the inclusion and use of these (and maybe the other species that were found) for the monitoring of secondary vegetation and quantification of succession time in LTF in northeastern Mexico. Further evidence of their usefulness as indicator species will contribute to the implementation of new and better conservation strategies, and to an efficient delimitation of natural protected areas which take into account the importance of secondary vegetation for species distribution.

## Conclusions

The study of effects of disturbance and secondary succession on species and communities is a key issue in ecology and conservation. In that sense, faunistic patterns of leaf beetles and their association to secondary succession are evaluated for first time in low thorn forest vegetation in northeastern Mexico. A highly fragmented landscape in early and intermediate successional stages, as well as the convergence of other vegetation communities, could be related to the high number of species found, due to a complex environmental heterogeneity. Overall, observed changes in communities were similar to those observed in other studies with leaf beetles in disturbance gradients. However, the inclusion of a seasonal effect results in some differences, depending on the evaluated parameter. Seasonal changes trigger differences in abundance, diversity, and species composition, but not in species richness, in each category and between categories. Major influence of seasonality occurred at intermediate successional categories, which could be due to the influence of intermediate disturbance hypothesis. Therefore, we point out the importance of evidence here obtained, since the influence of seasonal changes on successional trajectories is important for every biological taxon. Thus, accelerating climate change would exert modifications in the way communities are structured during secondary succession after disturbance, which is of major importance for species and ecosystem restoration. On the other hand, we propose that the use of several species of leaf beetles for monitoring secondary vegetation and quantifying the succession time of low thorn forest is feasible, at least in northeastern Mexico. However, further studies are necessary to assess the potential of these and other species of leaf beetles to be indicators.

## Appendix 1

**Table:** Taxonomic checklist of Chrysomelidae per season in each category of succession time, in a fragment of low thorn forest in northeastern Mexico. Key: 4y = 4 years of succession, 17y = 17 years of succession, 31y = 31 years of succession, CA = Conserved areas.

Species	Rainy season				Dry season			
	4y	17y	31y	CA	4y	17y	31y	CA
CRIOCERINAE Latreille, 1807								
Tribe Lemini Heinze, 1962								
*Lema* sp. 1								1
*Lema* sp. 2							1	
*Oulema* sp. 1 *	1							
*Neolema* sp. 1	1							
CASSIDINAE Gyllenhal, 1813								
Tribe Chalepini Weise, 1910								
*Baliosus* sp. 1	2		4	3		2		
*Brachycorynapumila* Guérin-Méneville, 1844	33				30	8		
*Chalepusamabilis* Baly 1885								3
*Chalepusbellulus* (Chapuis, 1877)	3							
*Chalepusdigressus* Baly 1885				2				
*Heterispavinula* (Erichson, 1847)	21	50	23	7	15	43	21	
*Pentispadistincta* (Baly 1885)								2
*Sumitrosisinaequalis* (Weber, 1801)	1	1	4			2	7	
Tribe Ischyrosonychini Chapuis, 1875								
*Physonotaalutacea* Boheman, 1854		6	3			4		
Tribe Cassidini Gyllenhal, 1813								
*Agroiconotavilis* (Boheman, 1855)					1			
*Charidotellabifossulata* (Boheman, 1855)								1
*Charidotellasexpunctata* (Fabricius, 1781)	2			2			3	3
*Charidotisauroguttata* Boheman, 1855				4				
*Helocassisclavata* (Fabricius, 1798)	3	1		17	2	3	2	9
CHRYSOMELINAE Latreille, 1802								
Tribe Chrysomelini Latreille, 1802								
Subtribe Doryphorina Motschulsky, 1860								
*Calligraphaancoralis* Stål, 1860	10	1						
*Calligraphapiceicollis* Stål, 1859	1				2			
*Deuterocamptaatromaculata* Stål, 1859)		1	3	2			2	
Subtribe Chrysomelina Latreille, 1802								
*Plagioderasemivittata* Stål, 1860		1					3	
*Plagioderathymaloides* Stål, 1860	8	1	8		8		3	
GALERUCINAE Latreille, 1802								
Tribe Galerucini Latreille, 1802								
Group Coelomerites Chapuis, 1875								
*Coraiasubcyanescens* (Schaeffer, 1906)		3	1	1				
*Derospideaornata* (Schaeffer, 1905)			2					
*Miracesaeneipennis* Jacoby, 1888								1
Tribe Luperini Chapuis, 1875								
Subtribe Diabroticina Chapuis, 1875								
Group Diabroticites Chapuis, 1875								
*Diabroticalitterata* Sahlberg 1823	8			4	2			
Group Cerotomites Chapuis, 1875								
*Cyclotrypemafurcata* (Olivier, 1808)	31	32	3		7	20		
Tribe Alticini Newman, 1835								
*Acallepitrix* sp. 1		4	1	8		2		4
*Acallepitrix* sp. 2				5				
*Acrocyumdorsalis* Jacoby, 1885				11				4
*Alagoasabipunctata* (Chevrolat, 1834)		4	4	9				2
*Alagoasajacobiana* (Horn, 1889)	5		6	17		4		10
*Alagoasa* sp. 1						1		
*Alagoasa* sp. 2	2							
*Asphaera* sp. 1	7	1			3			
*Centralaphthonadiversa* (Baly, 1877)	280	1172	1261	167	103	240	217	18
*Chaetocnema* sp. 1	976	61			41	8		2
*Chaetocnema* sp. 2	1				3			2
*Dibolia* sp. 1				1				
*Disonychaglabrata* (Fabricius, 1781)	4	1		1				
*Disonycha* sp. 2					16			
*Disonychastenosticha* Schaeffer, 1931			2	1				
*Dysphenges* sp. 1	14	13	25	2			2	
*Epitrix* sp. 1	287	1	1	44	320	3		12
*Epitrix* sp. 2	16	3	3	25		4		21
*Epitrix* sp. 3	183	7		27	41		2	
*Epitrix* sp. 4	5	1		16	4			20
*Epitrix* sp. 5	1	2		4	2	2		8
*Heikertingerella* sp. 1				3				
*Longitarsus* sp. 1	32	10	10	40	7	4		8
*Macrohalticajamaicensis* (Fabricius, 1792)	1							
*Margaridisa* sp. 1	46		21	80	8	3	8	152
*Margaridisa* sp. 2								4
*Monomacrabumeliae* (Schaeffer, 1905)	4	3		3		2		
*Omophoitacyanipennis* (Fabricius, 1798)					3			
*Parchicola* sp. 1				11	2			6
*Parchicola* sp. 2	6							
*Parchicola* sp. 3				1				
*Syphrea* sp. 1	2			1				
*Syphrea* sp. 2								2
*Syphrea* sp. 3	3							
*Systena* sp. 1					1			
EUMOLPINAE Hope, 1840								
Tribe Eumolpini Hope, 1840								
Group Iphimeites Chapuis, 1874								
*Brachypnoea* sp. 1	4							
*Colaspismelancholica* Jacoby, 1881				3				
*Colaspis* sp. 1	1							
*Colaspistownsendi* Bowditch, 1921	20	1		5	15			
*Zenocolaspisinconstans* (Lefèvre, 1878)	3		2	2	3			
Tribe Typophorini Chapuis, 1874								
Group Typophorites Chapuis, 1874								
*Paria* sp. 1			1	1				
CRYPTOCEPHALINAE Gyllenhal, 1813								
Tribe Cryptocephalini Gyllenhal, 1813								
Subtribe Pachybrachina Chapuis, 1874								
*Pachybrachis* sp. 1	5	1			2	4		
*Pachybrachis* sp. 2	1				4			
*Pachybrachis* sp. 3	3	1					4	
*Pachybrachis* sp. 4	3	2			4		2	
*Pachybrachis* sp. 5	1							
*Pachybrachis* sp. 6	1							
Subtribe Cryptocephalina Gyllenhal, 1813								
*Cryptocephalusguttulatus* Olivier,1808	1							
*Cryptocephalustrizonatus* Suffrian, 1858	3				4			
*Diachus* sp. 1			2					
Tribe Clytrini Lacordaire, 1848								
Subtribe Clytrina Lacordaire, 1848								
*Anomoearufifronsmutabilis* (Lacordaire, 1848)	1	1						
*Smaragdinaagilis* (Lacordaire, 1848)					2			
Subtribe Ischiopachina Chapuis, 1874								
*Ischiopachysbicolorproteus* Lacordaire, 1848	5		1					
Subtribe Babiina Chapuis, 1874								
*Babiatetraspilotatexana* Schaeffer, 1933	10	3	6	1	4	2		
Subtribe Megalostomina Chapuis, 1874								
*Proctophana* sp. 1					1			
Tribe Fulcidacini Jakobson, 1924								
*Chlamisus* sp. 1	1							
*Diplacaspisprosternalis* (Schaeffer, 1906)	2	1				2		
